# Modified scanning electron microscopy reveals pathological crosstalk between endothelial cells and podocytes in a murine model of membranoproliferative glomerulonephritis

**DOI:** 10.1038/s41598-018-28617-1

**Published:** 2018-07-06

**Authors:** Md. Abdul Masum, Osamu Ichii, Yaser Hosny Ali Elewa, Teppei Nakamura, Yuki Otani, Marina Hosotani, Yasuhiro Kon

**Affiliations:** 1 Laboratory of Anatomy, Department of Basic Veterinary Sciences, Faculty of Veterinary Medicine, Hokkaido University, Sapporo, Japan; 2 Department of Anatomy, Histology and Physiology, Faculty of Animal Science and Veterinary Medicine, Sher-e-Bangla Agricultural University, Dhaka, Bangladesh; 3 Department of Histology, Faculty of Veterinary Medicine, Zagazig University, Zagazig, Egypt; 4 Section of Biological Safety Research, Chitose Laboratory, Food Research Laboratories, Chitose, Japan

## Abstract

This study evaluated endothelial cells and podocytes, both being primary components of the glomerular filtration barrier, in the progression of membranoproliferative glomerulonephritis (MPGN) using modified scanning electron microscopy (mSEM) analysis. BXSB/MpJ-*Yaa* model mice exhibited autoimmune-mediated MPGN characterised by elevated serum autoantibody levels, albuminuria, renal dysfunctional parameters, and decreased glomerular endothelial fenestrations (EF) and podocyte foot process (PFP) effacement with immune cell infiltration. Similar to transmission electron microscopy, mSEM revealed a series of pathological changes in basement membrane and densities of EF and PFP in BXSB/MpJ-*Yaa* compared with control BXSB/MpJ at different stages. Further, immunopositive area of endothelial marker (CD34), podocyte functional molecules (Nephrin, Podocin, Synaptopodin, and Wilms’ tumour 1 (WT1)), and vascular endothelial growth factor A (VEGF A) significantly decreased in the glomerulus of BXSB/MpJ-*Yaa* compared with BXSB at final stage. The indices of glomerular endothelial injuries (EF density and immunopositive area of CD34 and VEGF A) and podocyte injuries (PEP density and immunopositive area of podocyte functional molecules) were also significantly correlated with each other and with indices of autoimmune disease and renal dysfunction. Thus, our results elucidated the pathological crosstalk between endothelial cells and podocytes in MPGN progression and the usefulness of mSEM for glomerular pathological analysis.

## Introduction

The mammalian kidney is generally considered a non-regenerative organ. Recently, the incidence of kidney diseases, especially chronic kidney disease (CKD), has increased worldwide^1^. Therefore, it is considered a serious public health concern often associated with end-stage renal disease (ESRD) and cardiovascular disease. Importantly, the membranoproliferative glomerulonephritis (MPGN) is one of the prevalent primary contributors to CKD and is considered the third leading cause of ESRD among the primary glomerulonephritis^2,3^.

The adult kidney filters blood to eliminate toxins and metabolic wastes, maintains the balance of water, salts, and pH, absorbs minerals, produces urine, etc., to sustain life. The glomerular filtration barrier (GFB) consists of fenestrated glomerular endothelial cells (GECs), glomerular basement membrane (GBM), and podocytes, which play key roles to execute these functions. Defects in GFB integrity were observed during MPGN pathogenesis with leakage of plasma proteins, including albumin, finally leading to ESRD^4,5^. The occurrence of proteinuria was associated with podocytopathy and decreased podocyte number in MPGN^4^. Interestingly, it has been reported that, in healthy individuals, podocytes produced vascular endothelial growth factor (VEGF), essential for maintenance of adjacent endothelia and integrity of its fenestration^6,7^. Additionally, it has been reported that the glomerular endothelium was covered with a glycocalyx forming a permeability barrier preventing the development of proteinuria under normal healthy conditions^8,9^. Therefore, pathologies of GECs and their fenestration are associated with proteinuria and renal failure, which have been well characterized in preeclampsia and diabetes^10–12^. Moreover, an interaction between the endothelium and podocytes through VEGF has been reported in diabetes^13^. Furthermore, Haraldson *et al*. have reported that the GFB function was considered an integrated whole with cell-cell interaction, and any disruption of such interaction could affect the overall permeability^14^. However, limited information is available regarding the defective interactions between podocytes and glomerular endothelium during CKD pathogenesis, including MPGN revealed by combined ultrastructural and molecular approaches.

For the ultrastructural analysis of the glomerulus, scanning electron microscopy (SEM) and transmission electron microscopy (TEM) are useful. However, the ultrastructural quantitative analysis of the glomerulus, including enumeration of endothelial fenestrations (EFs) and podocyte foot process (PFP), is quite difficult, because SEM reveals only the surface of GECs or podocytes, and the area is limited for TEM observation. These methodological limitations are common in basic and clinical studies, and are associated with difficulty in biopsy-based diagnosis and ultrastructural analysis of the glomerulus. Interestingly, Koga *et al*. have performed SEM using a semi-thin section and reported that this method could aid visualising large areas of sections and obtain TEM-like images^15^. Therefore, in this study, we modified the previous SEM method to develop the so-called “mSEM” and applied this method for the analysis of glomerular ultrastructural pathology, especially focusing on MPGN.

In this study, we used BXSB/MpJ-*Yaa* (*Yaa*) mice as a murine model of MPGN. Mice of this strain develop systemic autoimmune disease characterised by increased serum autoantibody titres, autoreactive B cells, vasculitis, and podocyte injury^4^ with MPGN. *Yaa* carries a genetic mutation on the Y chromosome called “Y-linked autoimmune acceleration” (*Yaa)*, and male mice develop more severe MPGN than female mice due to the *Yaa* mutation^16–19^. The present study clearly revealed pathological correlations between the glomerular endothelial and podocyte injury in MPGN pathogenesis through a unique ultrastructural analysis and molecular quantitative analysis.

## Results

### Pathology of the MPGN model

**Figure 1:**
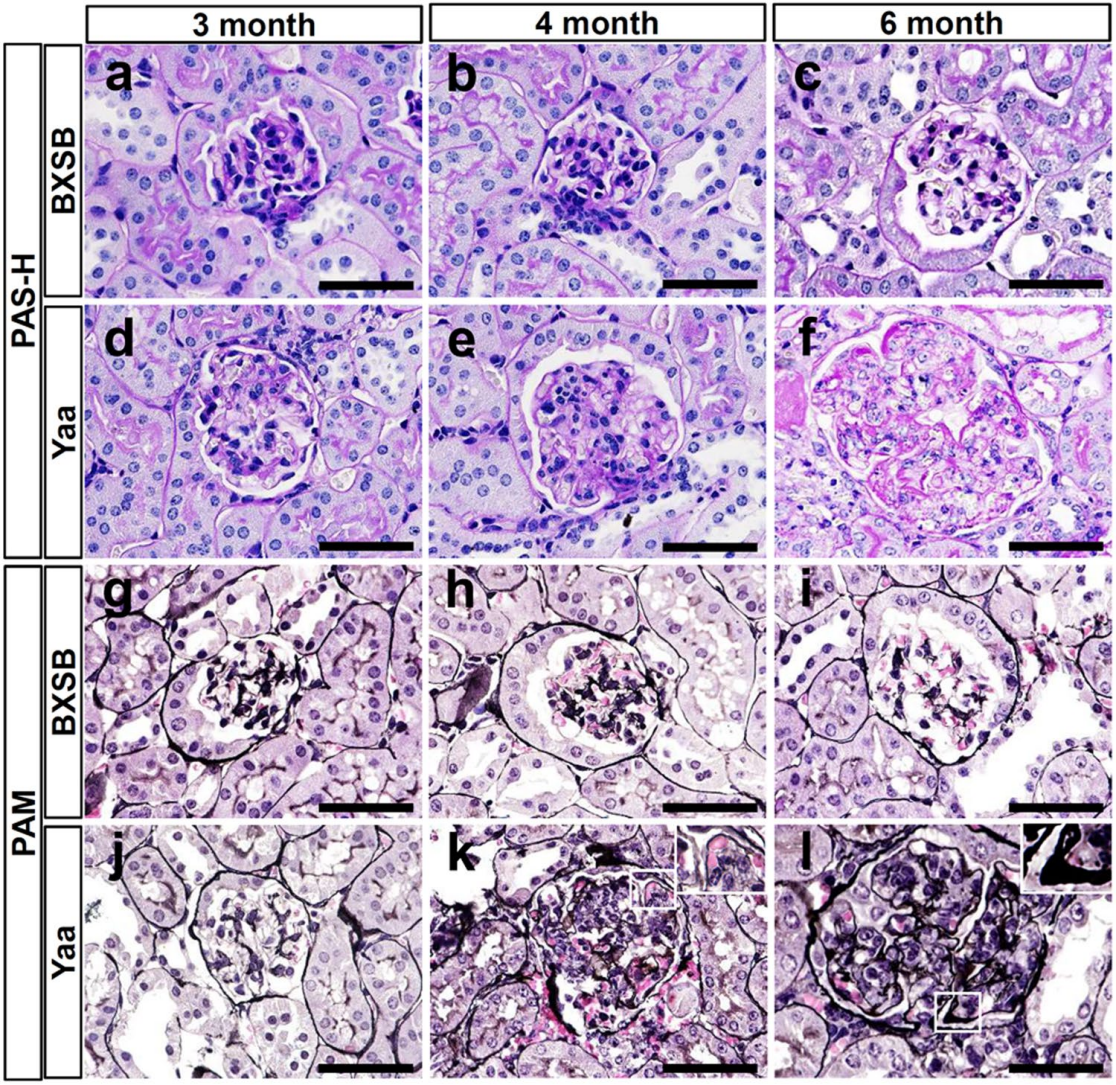
Autoimmune disease-mediated MPGN in *Yaa* mutants. (**a**–**f**) Glomerular histopathology, PAS-H staining. In BXSB mice, there are no glomerular lesions observed at 3, 4, and 6 months of age (**a**–**c**). In *Yaa* mutants, there are no lesions found in glomerulus at 3 months of age (**d**), but mild glomerulonephritis is found in glomerulus of 4-month-old *Yaa* mutants (**e**). MPGN characterised by increased glomerular size, cell number, and deposition of PAS-H-positive materials is clearly observed in glomerulus of *Yaa* mutants at 6 months of age (**f**). Bars = 50 µm. (**g** and **l**) Glomerular histopathology, PAM staining. GBM is normal in glomerulus of 3, 4, and 6 month-old BXSB mice (**g**–**i**). GBM is normal in glomerulus of 3-month-old *Yaa* mutants (**j**). Mild glomerular hypertrophy and thickening of GBM is found in *Yaa* mutants at 4 months of age (**k**). Glomerular hypertrophy, thickening, and wrinkling of GBM are clearly observed in *Yaa* glomerulus at 6 months of age (**l**). Bars = 50 µm. MPGN: membranoproliferative glomerulonephritis, *Yaa*: BXSB/MpJ-*Yaa*, BXSB: BXSB/MpJ, PAS-H: periodic acid Schiff-haematoxylin. PAM: periodic acid methenamine silver.

**Figure 2:**
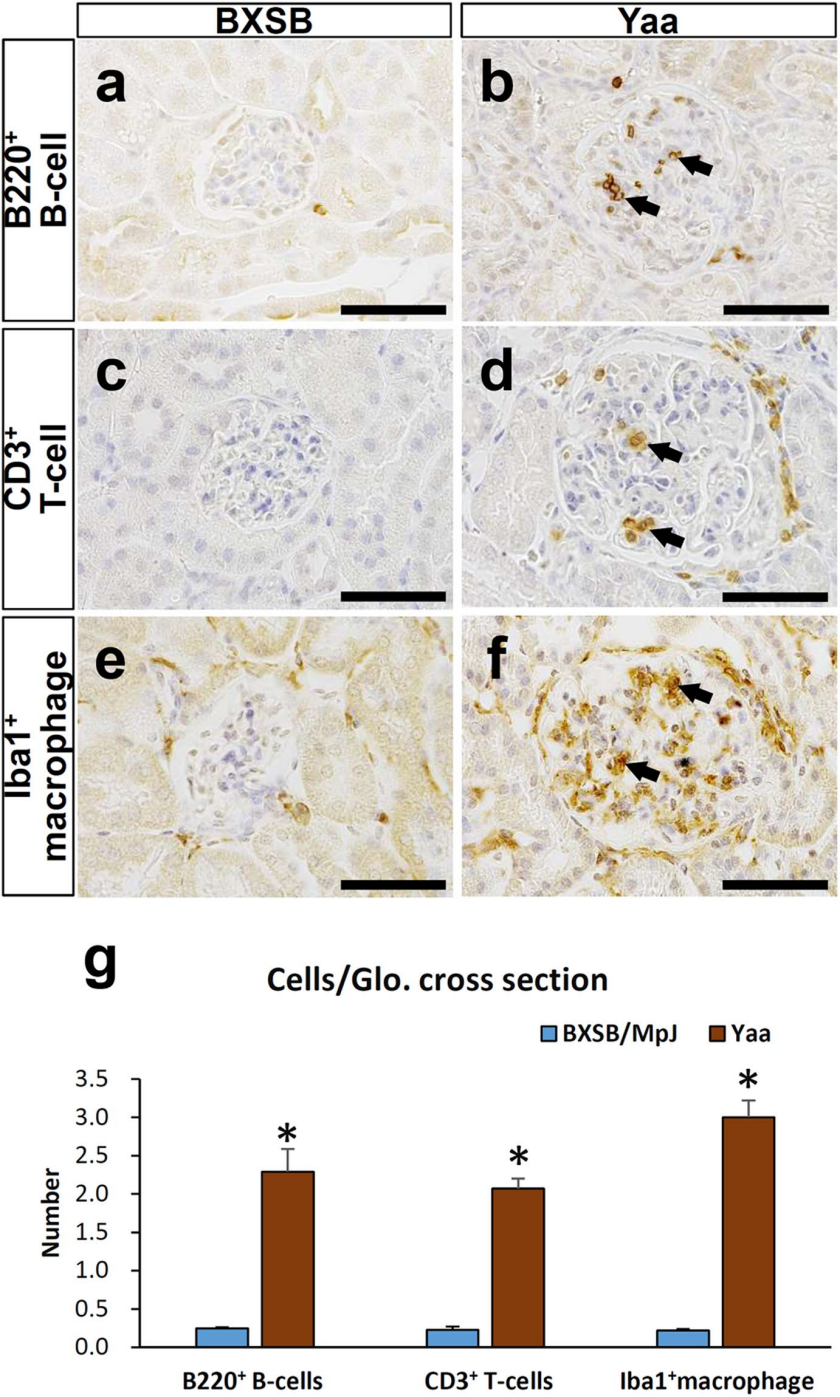
Infiltration of immune cells in the glomerulus. (**a–f**) Infiltration of immune cells in glomerulus, immunohistochemistry. B220^+^ B cells (**a**,**b**), CD3^+^ T cells (**c**,**d**), and Iba1^+^ macrophages (**e**,**f**) are abundant in the glomerulus of *Yaa* mutants (arrows) compared with BXSB mice at 6 months of age. Bars = 50 µm. (**g**) Quantification of immune cells in glomerulus of 4- and 6-month-old BXSB and Yaa mice. Values = mean ± standard error (s.e.). ^*^Significant difference from the control group, Mann-Whitney *U* test (*p* < 0.05). N = 4. *Yaa*: BXSB/MpJ-*Yaa*, BXSB: BXSB/MpJ, Glo.: Glomerulus.

Glomerular histopathology was examined in kidney sections from *Yaa* mutant mice and their respective control BXSB mice at 3, 4, and 6 months of age (Fig. 1). In BXSB mice, no glomerular lesion was observed in all ages examined (Fig. 1a–c). However, *Yaa* mutants showed specific lesion development depending on age. *Yaa* mutants at 3 months of age did not show any lesions, but some of them developed mild glomerulonephritis at 4 months of age (Fig. 1d,e). *Yaa* mutants at 6 months of age clearly developed MPGN characterised by glomerular hypertrophy, increased glomerular cell number, and periodic acid Schiff-haematoxylin (PAS-H)-positive material depositions in the mesangial area (Fig. 1f). There was no thickening of GBM in all ages of BXSB glomerulus (Fig. 1g–i). Though mild GBM thickening was observed in *Yaa* glomerulus at 4 months of age (Fig. 1k), it was more thickened and wrinkled in 6-month-old *Yaa* mutants (Fig. 1l). Other histopathological and clinical parameters were also estimated at 6 months of age as *Yaa* mutants showed clear lesions at this age. *Yaa* mutants at 6 months of age displayed significantly higher values in glomerular size and cell number (Supplementary Table 1), an index for systemic autoimmune abnormality (serum anti- double stranded DNA (dsDNA) antibody (ab) level), and renal functional indices (urinary albumin-to-creatinine ratio (uACR), serum blood urea nitrogen, serum creatinine (sCr) and (sBUN) (Supplementary Table 1). These data indicate the development of autoimmune-mediated MPGN at 6 months of age in *Yaa* mutants. Immunohistochemistry for B220, CD3, and Iba1 to detect B cells, T cells, and macrophages, respectively, revealed that they were present in the glomerulus of *Yaa* mutants at 6 months of age compared with BXSB mice (Fig. 2a–f), and these quantitative results were significantly higher in the former (Fig. 2g) than in BXSB mice.

### Loss of glomerular EF with podocyte injury examined through mSEM method in the MPGN model

**Figure 3:**
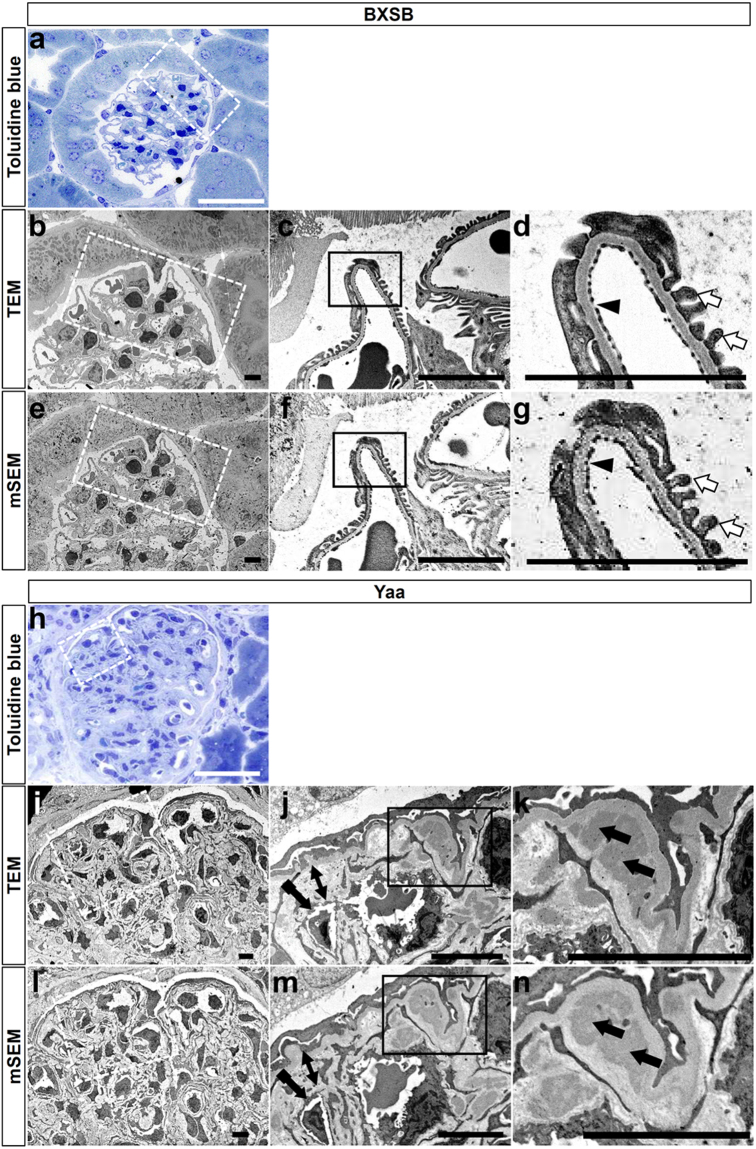
Ultrastructures of glomerulus examined by TEM and mSEM in BXSB and *Yaa* mice. (**a**) Glomerulus in BXSB mice, toluidine blue staining. (**b–d**) Glomerulus in BXSB mice, TEM. (**e–g**) Glomerulus in BXSB mice, mSEM. Dashed box area shows same location using different techniques (**a**,**b**,**e**). Normal EF (arrowhead) and PFP (white arrow) are clearly observed using TEM (**c**,**d**) and mSEM (**f**,**g**). Box area in panels (c) and (f) is magnified in panels (d) and (g), respectively. Bars = 5 µm. (**h**) Glomerulus in Yaa mutants, toluidine blue staining. (**i–k**) Glomerulus in Yaa mutants, TEM. (**l–n**) Glomerulus in Yaa mutants, mSEM. The dashed area shows the same location using different techniques. GBM widening (two headed arrows), endothelial thickening with loss of EF (tailed arrows), and deposition of electron-dense materials in GBM (black arrows) are clearly visible using both TEM (**j**,**k**) and mSEM (**m**,**n**). Box area in panels (j) and (m) is magnified in panels (k) and (n), respectively. Bars = 5 µm. *Yaa*: BXSB/MpJ-*Yaa*, BXSB: BXSB/MpJ, TEM: transmission electron microscopy, mSEM: modified scanning electron microscopy, PFP: podocyte foot process, EF: endothelial fenestration and GBM: glomerular basement membrane.

We modified and applied the previously described method^15^ for quantitative analysis of EF and PFP, and this was the first attempt at applying mSEM for pathological analysis (Fig. 3). Firstly, we could examine the same area of normal glomerulus in BXSB mice using light microscopy (Fig. 3a), TEM (Fig. 3b–d), and mSEM (Fig. 3e–g). EFs and PFPs were clearly observed through both TEM (Fig. 3c,d) and mSEM (Fig. 3f,g). Furthermore, we also examined the glomerulus of *Yaa* mutants (Fig. 3h–n). Endothelial cell hypertrophy with loss of EF and thickened GBM with electron-dense deposits was clearly visible in *Yaa* mutants using both TEM (Fig. 3i–k) and mSEM (Fig. 3l–n), indicating the usefulness of mSEM to examine the glomerular ultrastructure, similar to TEM.

**Figure 4:**
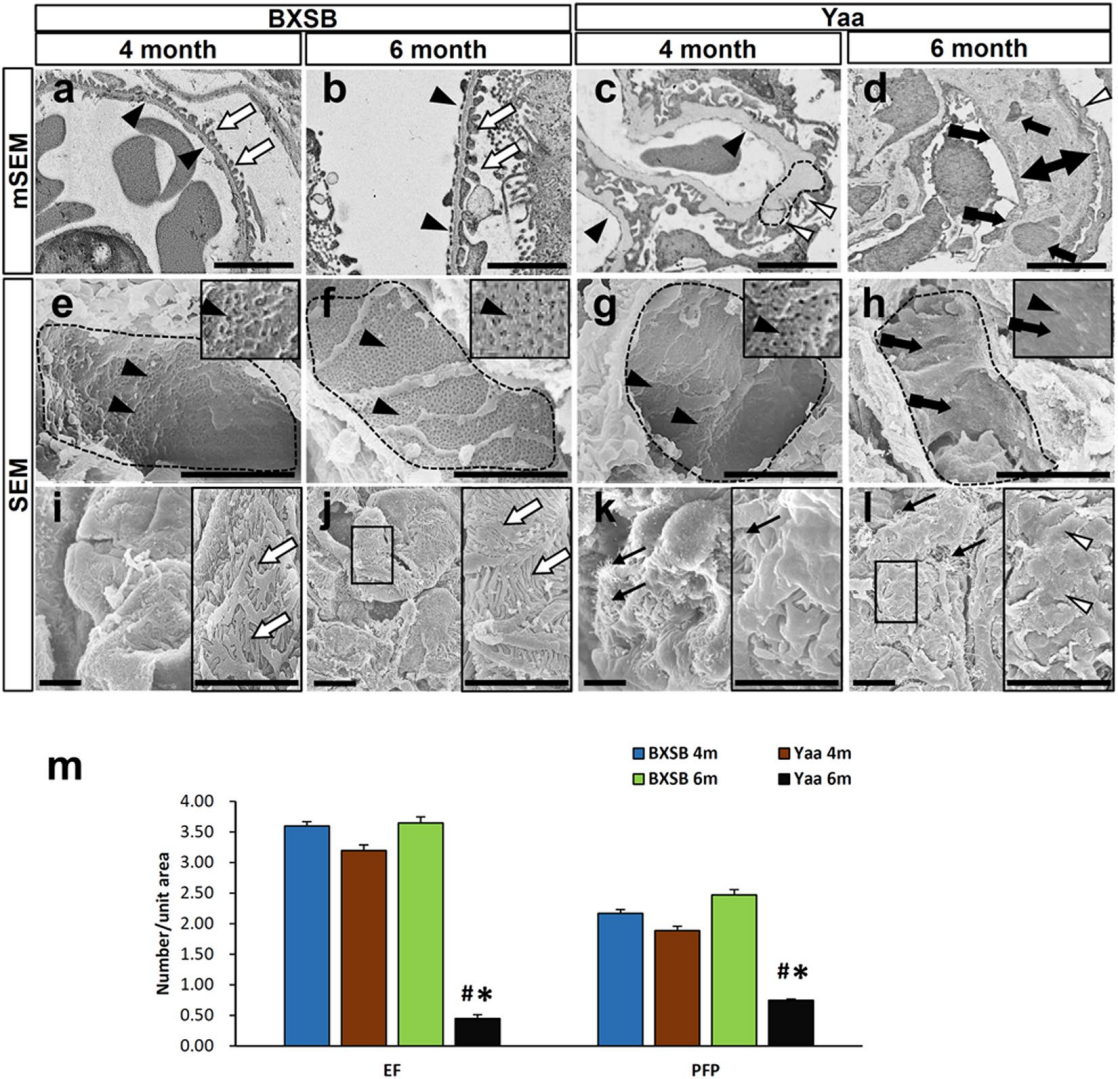
Injury of glomerular capillary and podocyte in *Yaa* mutants. (**a–d**) Glomerular capillary and podocyte injury, mSEM. There are well-distributed glomerular capillary EFs (arrowheads) and PFPs (white arrows) in BXSB mice at 4 and 6 months of age (**a**,**b**). Normal glomerular capillary EFs (arrowheads), mild PFPs (white arrowheads), and GBM thickening with hump (dashed area) are found in glomerulus of *Yaa* mutants at 4 months of age (**c**). GBM thickening (two headed arrows) with electron dense materials deposits (arrows), thickened endothelial cytoplasm with loss of EFs (tailed arrows), and PFP effacement (white arrowheads) are found in *Yaa* mutants at 6 months of age (**d**). Bars = 5 µm. (**e–l**) Injury of capillary and podocyte, SEM. There are normal glomerular capillary EFs (arrowheads) in BXSB mice at 4 and 6 months of age (**e**,**f**) and in *Yaa* mutants at 4 months of age (**g**), but loss of EF number (tailed arrows) is observed in glomerular capillary of *Yaa* mutants at 6 months of age (**h**). There are normal PFPs (white arrows) in BXSB mice at 4 and 6 months of age (**i**,**j**). *Yaa* mutants at 4 months of age show microvillus-like structure protruding from podocyte and mild PFPs effacement (**k**), and *Yaa* mutants at 6 months of age show numerous microvillus-like structures and severe PFP effacement (white arrowheads) (**l**). Bars = 5 µm. (**m**) Number of EFs and PFPs in 4- and 6-month-old BXSB and Yaa glomerulus. Values = mean ± standard error (s.e.) Values = mean ± standard error (s.e.). ^#^Significant difference from the control at the same age, Mann-Whitney *U* test (p < 0.05). ^*^Significant difference from other groups, Kruskal-Wallis test followed by Scheffe’s method (^*^p < 0.05, ^**^p < 0.01). N = 4. *Yaa*: BXSB/MpJ-*Yaa*, BXSB: BXSB/MpJ, mSEM: modified scanning electron microscopy, SEM: scanning electron microscopy, GBM: glomerular basement membrane, EFs: endothelial fenestrations, and PFPs: podocyte foot processes.

Figure 4 shows the ultrastructural features of EF and PFP using mSEM and SEM. mSEM in BXSB mice revealed that EFs and PFPs clearly lined the GBM in all ages examined (Fig. 4a,b). *Yaa* mutants showed normal endothelium with fenestrations at 4 months of age, but slight PFP effacement was observed at this stage (Fig. 4c). However, in *Yaa* mutants at 6 months of age, thickened GBM with electron-dense deposits was lined by hypertrophied endothelial cells with loss of EF and injured podocytes with PFP effacement (Fig. 4d). This is consistent with the results obtained using SEM, wherein BXSB glomerular capillary endothelium contained numerous EFs at all stages (Fig. 4e,f). However, *Yaa* mutants showed normal glomerular EFs and loss of EFs at 4 and 6 months of age, respectively (Fig. 4g,h). BXSB mice showed normal PFPs at all examined stages (Fig. 4i,j), but *Yaa* mutants at 4 months of age showed microvillus-like structure protruding from podocytes and mild PFP effacement (Fig. 4k). Numerous microvillus-like structures and PFPs effacement was observed in *Yaa* mutants at 6 months of age (Fig. 4l). Quantification analysis by mSEM revealed that the number of EFs and PFPs was significantly decreased in *Yaa* mutants compared to in BXSB mice (Fig. 4m) at 6 months of age.

### Downregulation of glomerular capillary endothelium marker, podocyte slit diaphragm molecules, podocytes, and VEGF A in the glomerulus of the MPGN model

**Figure 5:**
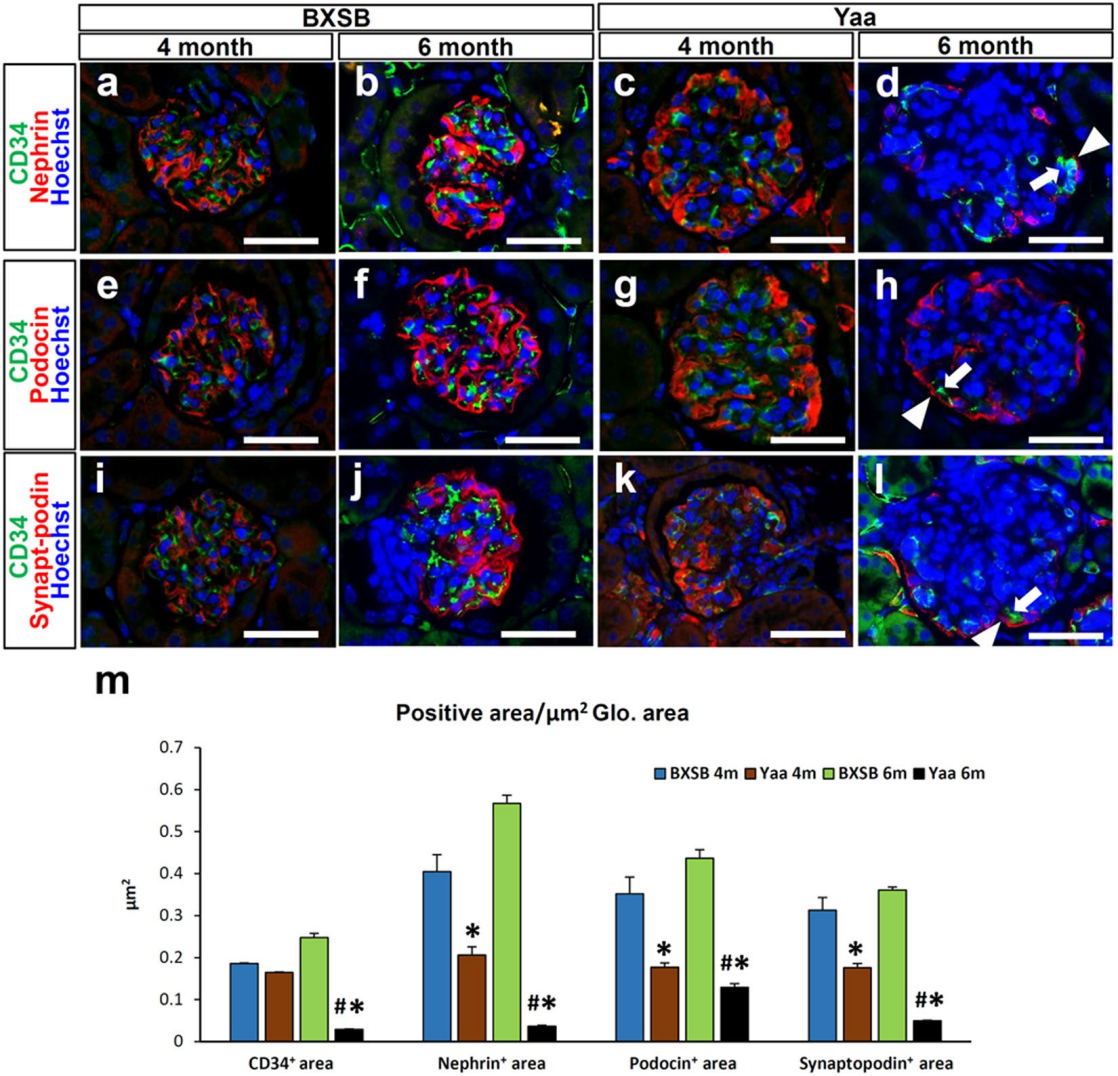
Loss of glomerular capillary and podocyte function molecules in *Yaa* mutants. (**a–l**) Endothelial cell marker (CD34) and podocyte function molecules (Nephrin, Podocin, and Synaptopodin), immunofluorescence. CD34-, Nephrin-, Podocin- and Synaptopodin-immunopositive areas are clearly visible in BXSB mice (**a**,**b**,**e**,**f**,**i**,**j**), but these are faint at the centre of glomerulus of *Yaa* mutants at 4 months of age (**c**,**g**,**k**) and lost in glomerulus of *Yaa* mutants at 6 months of age (**d,h**,**l**). Positive reactions for Nephrin, Podocin, and Synaptopodin (**d**,**h**,**l**, arrowheads) remain in the same areas as CD34^+^ capillary in *Yaa* mutants (arrows). Bars = 50 µm. (**m**) Immunopositive area for CD34, Nephrin, Podocin, and Synaptopodin in 4- and 6-month-old BXSB and Yaa mice. Values = mean ± standard error (s.e.). ^#^Significant difference from the control at the same age, Mann-Whitney *U* test (p < 0.05). ^*^Significant difference from other groups, Kruskal-Wallis test followed by Scheffe’s method (^*^p < 0.05, ^**^p < 0.01). N = 4. *Yaa*: BXSB/MpJ-*Yaa*, BXSB: BXSB/MpJ, Glo.: Glomerulus.

We next examined the expression of the capillary endothelium marker (CD34) and podocyte slit diaphragm molecules (Nephrin, Podocin, and Synaptopodin) in the glomerulus of BXSB and *Yaa* mice (Fig. 5a–l). CD34^+^ reactions were detected in the glomerular and tubulointerstitial capillary in the BXSB kidney using immunofluorescence staining (Fig. 5a,b,e,f,i,j). *Yaa* mutants at 4 months of age also showed normal CD34^+^ expression (Fig. c,g,k); however, glomerular capillaries were faint in the *Yaa* glomerulus at 6 months of age (Fig. 5d,h,l). Furthermore, linier positive reactions for podocyte slit diaphragm molecules (Nephrin, Podocin, and Synaptopodin) were clearly observed along with the glomerular capillary rete in BXSB mice at 4 and 6 months of age (Fig. 5a,b,e,f,i,j). Slit diaphragm molecules expression tended to be faint at the centre of glomerulus in the 4-month-old *Yaa* glomerulus, but they were faint throughout the glomerulus and tended to be localised only at the peripheral area in the *Yaa* glomerulus at 6 months of age (Fig. 5d,h,l). Importantly, podocyte function molecules were retained in those areas where CD34^+^ capillaries existed in *Yaa* mutants. Note that, there was normal expression of CD34^+^ and slit diaphragm molecule protein in juvenile (3 months) BXSB and *Yaa* mice (Supplementary Fig. 1). For quantification analysis, the immunopositive areas for Nephrin, Podocin, and Synaptopodin were decreased in 4- month-old *Yaa* mutants compared to in BXSB mice. However, the immunopositive areas for CD34, Nephrin, Podocin, and Synaptopodin in the glomerulus significantly were significantly decreased in *Yaa* mutants mice at 6 months of age (Fig. 5m).

**Figure 6:**
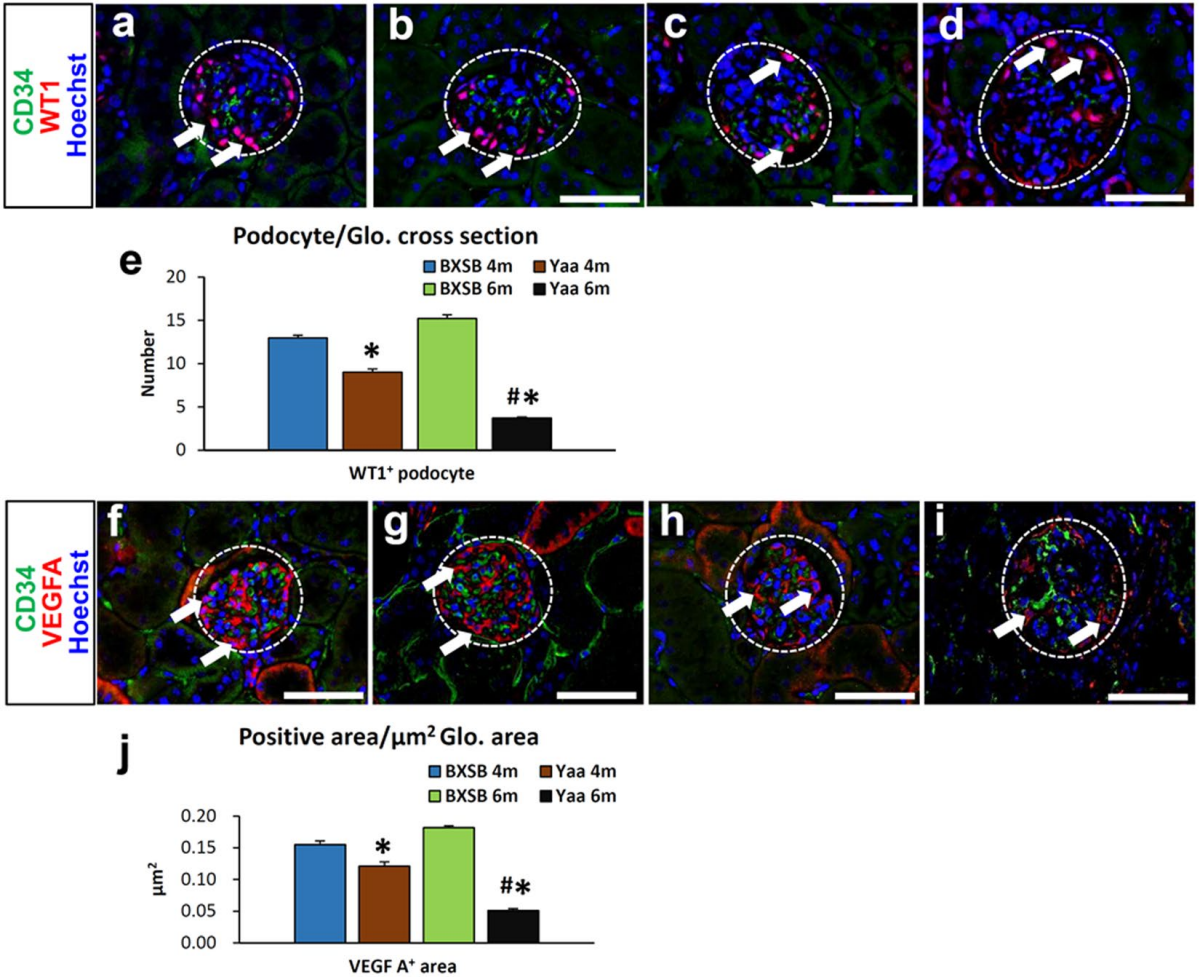
Loss of podocyte and VEGF A in *Yaa* mutants. (**a–d**) CD34+ endothelium and WT1+ podocytes, immunofluorescence. CD34^+^ capillary and WT1^+^ podocytes are well distributed in BXSB mice at 4 and 6 months of age (**a**,**b**). In *Yaa* mutants, WT1^+^ podocytes decrease at 4 months of age (**c**) but sharply decrease at 6 months of age (**d**). Bars = 50 µm. (**e**) Number of WT1+ podocytes in 6-month-old BXSB and Yaa mice. Values = mean ± standard error (s.e.). ^#^Significant difference from the control at the same age, Mann-Whitney U test (p < 0.05). ^*^Significant difference from other groups, Kruskal-Wallis test followed by Scheffe’s method (^*^p < 0.05, ^**^p < 0.01). N = 4. (**f–i**) CD34+ endothelium and VEGF A+ area, immunofluorescence. Normal distribution of CD34- and VEGF A^+^ area is observed in BXSB mice at 4 and 6 months of age (**f**,**g**). In *Yaa* mutants, VEGF A^+^ areas decrease at 4 and 6 months of age (**i**). Bars = 50 µm. (**j**) Measurement of VEGF A+ area in 4- and 6-month-old BXSB and Yaa mice. Values = mean ± standard error (s.e.). ^#^Significant difference from the control at the same age, Mann-Whitney *U* test (p < 0.05). ^*^Significant difference from other groups, Kruskal-Wallis test followed by Scheffe’s method (^*^p < 0.05, ^**^p < 0.01). N = 4. *Yaa*: BXSB/MpJ-*Yaa*, BXSB: BXSB/MpJ. WT1: Wilms’ tumour 1, and VEGF A: vascular endothelial growth factor A, Glo.: Glomerulus.

Wilms’ tumour 1 (WT1)^+^ podocytes were uniformly distributed throughout the BXSB glomerulus at 4 and 6 months of age (Fig. 6a,b). *Yaa* mutants at 4 months of age showed decreasing tendency for WT1^+^ podocytes (Fig. 6c); however, few podocytes were retained in *Yaa* mutants at 6 months of age (Fig. 6d). The number of podocytes also significantly decreased in the glomerulus of *Yaa* mutants compared to in BXSB mice at 4 and 6 months of age (Fig. 6e). VEGF A produced by podocytes in the glomerulus is responsible for the maintenance of the adjacent endothelium^20^. We observed a VEGF A positive reaction in the glomerulus of BXSB mice at all stages (Fig. 6f,g). However, weakly positive results were observed in *Yaa* mutants at 4 and 6 months of age (Fig. 6h,i). Note that, there was normal expression of WT1^+^ and VEGF A protein in juvenile (3 months) BXSB and *Yaa* mice (Supplementary Fig. 1). The immunopositive area of VEGF A also significantly decreased in the glomerulus of *Yaa* mutants compared to in BXSB mice at 4 and 6 months of age (Fig. 6j).

### Pathological correlation between histopathological and clinical parameters

The immunepositive area of CD34^+^ was correlated with the VEGF A^+^ and Podocin^+^ area whereas the number of EF was correlated only with the WT1^+^ podocyte number at 4 months of age (Supplementary Fig. 2). The immunopositive area of CD34^+^ and EF number was significantly correlated with all examined values, except for sCr (Table 1). The PFP number and VEGF A^+^ areas were also significantly correlated with all examined values, except for sCr (Table 1).

**Table 1:** Summary of correlation analysis

Groups	Parameters	CD34^+^ glomerular capillary		EF number		VEGF A^+^ glomerular area		PFP number		B cell		T cell		Macrophage	
		*ρ*	*p*	*ρ*	*p*	*ρ*	*p*	*ρ*	*p*	*ρ*	*p*	*ρ*	*p*	*ρ*	*p*
Endothelial cells	CD34	1.000	—	0.982**	<0.001	0.963**	<0.001	0.981**	<0.001	−0.927**	0.001	−0.972**	<0.001	−0.969**	<0.001
	EF	0.982**	<0.001	1.000	—	0.958**	<0.001	0.979**	<0.001	−0.941**	<0.001	−0.974**	<0.001	−0.982**	<0.001
	VEGF A	0.963**	<0.001	0.958**	<0.001	1.000	—	0.991**	<0.001	−0.910**	0.002	−0.971**	<0.001	−0.961**	<0.001
Podocytes	PFP	0.981**	<0.001	0.979**	<0.001	0.985**	<0.001	1.000	—	−0.928**	0.001	−0.988**	<0.001	−0.976**	<0.001
	Nephrin	0.986**	<0.001	0.998**	<0.001	0.954**	<0.001	0.979**	<0.001	−0.933**	0.001	−0.978^**^	<0.001	−0.974**	<0.001
	Podocin	0.962**	<0.001	0.973**	<0.001	0.905**	0.002	0.954**	<0.001	−0.931^**^	0.001	−0.946^**^	<0.001	−0.960**	<0.001
	Synaptopodin	0.994**	<0.001	0.981**	<0.001	0.974**	<0.001	0.969**	<0.001	−0.930^**^	0.001	−0.980^**^	<0.001	−0.974**	<0.001
	WT1	0.988**	<0.001	0.990**	<0.001	0.979**	<0.001	0.985**	<0.001	−0.921^**^	0.108	−0.979^**^	<0.001	−0.971**	<0.001
Inflammatory cells	B-cell	−0.927**	0.001	−0.941**	<0.001	−0.910**	0.002	−0.928**	0.001	1.000	—	0.889^**^	0.004	−0.978**	<0.001
	T cell	−0.972**	<0.001	−0.974**	<0.001	−0.971**	<0.001	−0.986**	<0.001	0.889^**^	0.004	1.000	—	−0.956**	<0.001
	Macrophage	−0.969**	<0.001	−0.982**	<0.001	−0.961**	<0.001	−0.976**	<0.001	−0.978**	<0.001	−0.956**	<0.001	1.000	—
Glomerular morphology	Glo. size	−0.928**	0.001	−0.929**	0.001	−0.936**	0.001	−0.948**	0.001	0.937^**^	<0.001	0.953^**^	<0.001	−0.960**	<0.001
	Glo. cell	−0.969**	<0.001	−0.980**	<0.001	−0.967**	<0.001	−0.977**	<0.001	0.896^**^	0.003	0.976^**^	<0.001	−0.968**	<0.001
Renal function	sBUN	−0.845**	0.01	−0.824*	0.012	−0.795*	0.018	−0.835**	0.010	0.679	0.064	0.815^*^	0.014	0.724*	0.042
	sCr	−0.678	0.065	−0.700	0.053	−0.664	0.072	−0.689	0.059	0.646	0.084	0.615	0.105	0.695	0.0556
	uACR	−0.862**	0.006	−0.835**	0.010	−0.834*	0.010	−0.850**	0.008	0.642	0.074	0.899^**^	0.002	0.754*	0.031
Autoimmune abnormality	Anti-dsDNA ab	−0.937**	0.001	−0.946**	<0.001	−0.919**	0.001	−0.924**	0.001	0.964^*^	<0.001	0.929^**^	0.001	−0.768*	0.028

*p < 0.05 and **p < 0.01, Pearson’s rank correlation coefficient, N = 8. Cap.: Capillary, EF: Endothelial fenestration, PFP: Podocyte foot process, Glo.: Glomerulus, sBUN: Serum blood urea nitrogen, sCr: Serum creatinine, abs: Antibody, uACR: Urine albumin-to-creatinine ratio, — Not applicable.

## Discussion

*Yaa* mutants (MPGN model mice) at 3 months of age showed a normal glomerulus. However, individual discrepancies were observed in lesion development at 4 months, and some showed only podocyte injury. *Yaa* mutants at 6 months of age developed clear lesions for endothelial cells and podocytes. Therefore, we examined the crosstalk between the endothelium and podocytes using model mice at different ages by mSEM to monitor disease progression.

Qualitative data for renal ultrastructures in pathological conditions are available; however, corresponding quantitative data are relatively scarce. Ultrastructural evaluation of the kidney by TEM in both basic and clinical studies, is crucial for definitively diagnosing glomerular diseases such as MPGN, membranous nephropathy, lupus nephritis, or minimal change disease in humans and animals^4,5,21^. However, TEM is complex and reveals only a small observation area. This makes the quantitative ultrastructure study more laborious and prolonged. Therefore, we modified the previously described SEM method^15^ and demonstrated its efficacy for renal pathological analysis for the first time. In healthy BXSB mice, glomerular EF and PFP were clearly observed using both TEM and mSEM (Fig. 3). Moreover, in *Yaa* mutants, clear glomerular lesions such as endothelial thickening with decreased EF, GBM widening, immune-complex depositions in GBM, and PFP effacement were detected using both TEM and mSEM (Fig. 3). Therefore, mSEM is an effective method to evaluate the glomerular ultrastructure in both basic and clinical studies.

The combinations of mSEM and immunostaining methods revealed that *Yaa* mutants had endothelial injury with EF loss and decreased CD34^+^ area in the glomerulus. Glomerular capillary endothelium is highly flattened, fenestrated, and has glycocalyx covering the luminal surface, essential for formation of glomerular ultrafiltrate and permeability barrier^13,22,23^. Therefore, endothelial cell injury with morphological alternations is crucial for the development of glomerular lesions and tubulointerstitial lesions in the kidney^5,24,25^. In this study, the EF number and CD34^+^ area were normal at 4 months of age in the *Yaa* mutants but decreased and strongly correlated with the histopathological indices at 6 months of age (Fig. 5 and Table 1). Thus, autoimmune disease-mediated inflammation is a key factor in the pathogenesis of endothelial injuries as many infiltrated B-, T-cells, and macrophages were observed in the Yaa glomerulus (Fig. 2). A previous study also reported B-cells, T-cells, and secretion of pro-inflammatory cytokines including tumour necrosis factor α and interleukin 6 contribute to glomerular lesions in lupus-prone mice^26,27^. Moreover, macrophages and their products resulted in glomerulosclerosis^28^.

mSEM revealed PFP effacement at 4 and 6 months of age (Fig. 4). Similarly, the immunopositive area for podocyte functional molecules was decreased at 4 and 6 months (Figs 5 and 6). These data indicate that mSEM is useful for analysing the glomerular ultrastructure in the disease state.

Podocytes are specialised perivascular cells that maintain the GFB through formation of podocyte function molecules and interaction with adjacent endothelium^6,29–31^. The impaired expression of podocyte function molecules are primary contributor to the development of podocyte injury and subsequent albuminuria in several glomerular diseases^32–37^. In the present study, the immunopositive area for podocyte function molecules was decreased at 4 months of age in *Yaa* mutants. However, the decreased Podocin^+^ area was correlated with a normally distributed CD34^+^ area (Supplementary Table 2). This indicates that Podocin maintained the CD34^+^ area at this stage. Additionally, a decreasing number of WT1^+^ podocyte was correlated with the EF number at 4 months of age (Supplementary Table 2). This suggests that a profound loss of podocyte would be related to EF loss. Importantly, in 6 month-old *Yaa* mutants, podocyte function molecules and WT1^+^ podocyte remained in the areas where CD34^+^ capillaries were existed, and podocyte injury indices were strongly correlated with glomerular capillary injury in *Yaa* mutants (Figs 5 and 6, Table 1). Interestingly, other studies have showed imbalanced production of endothelial nitric oxide, activated protein C, and endothelin type 1 receptor from glomerular endothelium-mediated podocyte injury in glomerulonephritis^38,39^. These results strongly suggest the functional correlation between glomerular endothelium and podocytes.

The formation of the glomerular capillary EF critically depends on VEGF A from differentiated podocytes^20^. Eremina *et al*. have reported that podocyte-specific deletion of a single allele of VEGF A resulted in loss of endothelial differentiation without any fenestration^40^. Interestingly, VEGF signalling was not involved in the endothelial-podocyte interaction until advanced podocyte injury was established^39^. In this study, we observed uniform expression of VEGF A at all ages of BXSB mice. VEGF A^+^ area began to decrease in 4-month-old *Yaa* mutants glomerulus, but was drastically reduced at 6 months (Fig. 6). Furthermore, the VEGF A^+^ area was strongly correlated with podocyte and glomerular endothelial injury only at 6 months (Table 1). Therefore, a severely injured and/or reduced number of podocytes produced a lower quantity of VEGF A. This decreased the bioavailability of VEGF A in the glomerulus, resulting in glomerular endothelial injury and EF loss.

**Figure 7:**
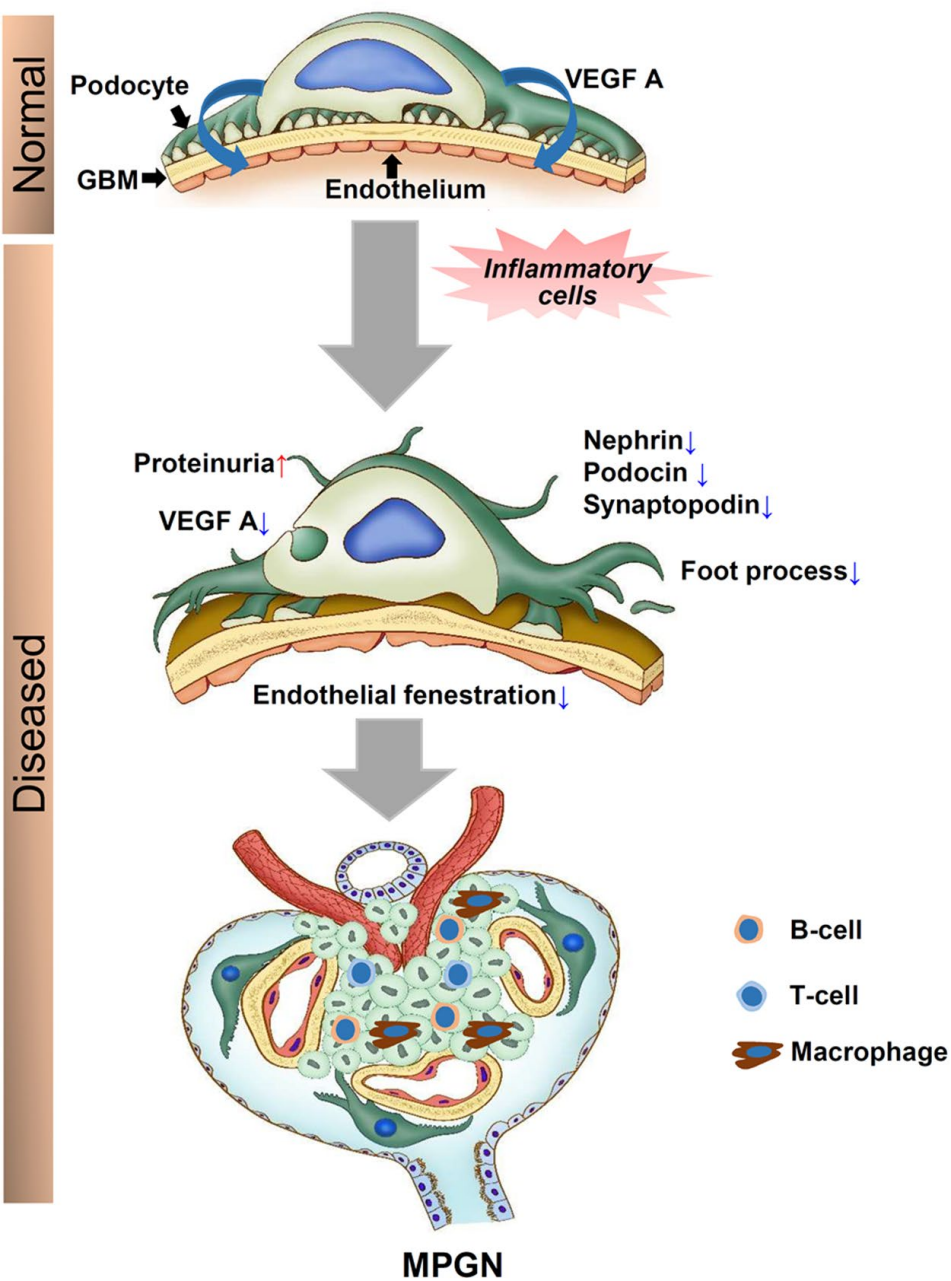
Pathological alternations of GFB in the development of MPGN. GFB consists of glomerular endothelium, GBM, and podocyte. Podocyte produces VEGF A responsible for maintenance of adjacent endothelium. Increase in inflammatory cells results in podocyte injury, loss of its functional molecules, and proteinuria. Finally, injury and subsequent loss of podocyte results in decreased production of VEGF A that aggravates the endothelial damage and loss of fenestration. Crosstalk between endothelium and podocytes occurs in order to develop MPGN. GFB: glomerular filtration barrier, GBM: glomerular basement membrane, and MPGN: membranoproliferative glomerulonephritis.

The GFB is a highly complex biological structure maintained by various physiochemical molecules and signalling pathways among its three core components^41^. We summarised pathological events of GFB occurring in the MPGN model (Fig. 7). Briefly, endothelial cells are dependent on podocytes for VEGF A to maintain their morphology and fenestration. However, inflammatory cells cause podocyte injury resulting in PFP effacement, downregulation of slit diaphragm molecules, and even loss of whole podocytes. This injury and loss of podocytes causes proteinuria. Simultaneously, VEGF A production drastically reduced from the severely injured podocytes, which aggravates endothelial damage resulting in loss of fenestration. In addition, imbalanced production of signalling molecules from injured endothelium also affects podocytes. Hence, glomerular capillary endothelial and podocyte injury mutually disrupts GFB and leads to MPGN pathogenesis.

## Methods

### Ethical statements

All animal experiments were approved by the Institutional Animal Care and Use Committee of the Faculty of Veterinary Medicine, Hokkaido University (approval No. 13-0032, 16-0124). The authors adhered to approved Guide for the Care and Use of Laboratory Animals of Hokkaido University, Faculty of Veterinary Medicine (approved by the Association for Assessment and Accreditation of Laboratory Animal Care International).

### Experimental animal and housing

Male BXSB/MpJ (BXSB) and BXSB/MpJ-*Yaa* (*Yaa*) mice were purchased from Japan SLC Inc. (Hamamatsu, Japan). *Yaa* mutants were used as autoimmune MPGN model mice and age-matched BXSB mice served as the control. All mice were maintained in specific pathogen-free conditions in a 1:1 light-dark condition. Food and water were provided *ad libitum* to experimental mice.

### Sample preparation

Mice were deeply anaesthetised with a mixture of 0.3 mg/kg medetomidine (Kyoritsu Seiyaku, Tokyo, Japan), 4 mg/kg midazolam (Astellas Pharma, Tokyo, Japan), and 5 mg/kg butorphanol (Meiji Seika Pharma, Tokyo, Japan), and urine was collected through urinary bladder puncture. Mice were euthanised by exsanguination from femoral artery. Blood and kidneys were collected for serological and histopathological analysis, respectively.

The harvested kidneys were cut into small slices and fixed with 10% neutral buffer formalin (NBF), 4% paraformaldehyde (PFA), or 2.5% glutaraldehyde (GTA) in 0.1 M phosphate buffer (PB) for histopathological analysis, immunofluorescence staining, and ultrastructural analysis, respectively.

### Serum and urine analysis

Serum levels of anti-dsDNA ab in all mice were measured using Mouse Anti-dsDNA Ig’s (Total A + G + M) ELISA kit (Alpha Diagnostic International, San Antonio, TX, USA) to evaluate the autoimmune condition. sBUN and sCr levels in all mice were measured using Fuji Drichem 7000 v (Fujifilm, Tokyo, Japan) to examine kidney function. uACR was determined using Albuwell M and Creatinine Companion kits (Exocell, Philadelphia, PA, USA).

### Histopathological examination

Paraffin blocks of kidney specimens fixed with NBF were cut at a thickness of 2 μm and stained with periodic acid Schiff-haematoxylin (PAS-H) and periodic acid methenamine silver (PAM) to examine histopathology of the glomerulus. Immunodetection of cell markers for B cells (B220), T cells (CD3), capillary endothelial cells (CD34), macrophages (Iba1), podocytes (Nephrin, Podocin, Synaptopodin, and WT1), and VEGF A was performed. Staining conditions are listed in Supplementary Table 3. Briefly, deparaffinised kidney sections were subjected to antigen retrieval. Thereafter, slides were submerged in methanol containing 3% H_2_O_2_ for 20 min at 25 °C and blocked with normal goat or donkey serum. Sections were incubated with primary antibody overnight at 4 °C. After washing in phosphate-buffered saline (PBS), sections were incubated with respective secondary antibody or Alexa Fluor 488-labelled donkey anti-rat IgG (Life Technologies, Tokyo, Japan) or Alexa Fluor 546-labelled donkey anti-rabbit antibody (Life Technologies) at 25 °C for 30 min and then washed with PBS. For immunofluorescence, the tissue sections were incubated with Hoechst 33342 (1: 200; Dojingo, Kumamoto, Japan) for nuclear staining at room temperature for 5 min and examined under All-in-One Fluorescence Microscope BZ-X710 (Keyence, Osaka, Japan). For immunohistochemistry, the sections were incubated with biotinylated secondary antibody and then streptavidin-horseradish peroxidase (SABPO kit; Nichirei, Tokyo, Japan) for 30 min followed by incubation with 3,3-diaminobenzidine tetrahydrochloride-H_2_O_2_ solution. Finally, the sections were counterstained with haematoxylin and dehydrated with ascending grades of alcohols.

### Electron microscopy

We modified the method described by Koga *et al*. to obtain TEM-like images using SEM (Supplementary Fig. 2)^15^. Briefly, small pieces of kidney sections were fixed with 2.5% GTA in 0.1 M PB for 4 h at 4 °C followed by post-fixation with 1% osmium tetroxide (OsO_4_) in 0.1 M PB for 2 h. Then, specimens were dehydrated with ascending grades of alcohol and embedded in epoxy resin (Quetol 812 Mixture; Nisshin EM, Tokyo, Japan). The Epon blocks were cut at a thickness of 500 nm. Semi-thin sections were mounted on cover glass slides and incubated at 60 °C for 30 min after staining with toluidine blue. The sections were stained with uranyl acetate and lead citrate for 20 min and 15 min, respectively. The section containing cover glass was mounted on the specimen stub using two-sided adhesive and sputter-coated for 60 s with Hitachi E-1030 ion sputter coater (Hitachi, Tokyo, Japan), and then examined using an S-4100 SEM (Hitachi) with an accelerating voltage of 4 kV.

For routine SEM, small pieces of GTA-fixed kidney were treated with tannic acid and post-fixed with 1% osmium tetroxide for 1 h. The specimens were dehydrated with grades of alcohol, transferred into 3-methylbutyl acetate, and finally dried with HCP-2 critical point dryer (Hitachi, Tokyo, Japan). The specimens were then examined with an S-4100 SEM. For TEM examination, small pieces of kidney were pre-fixed with 2.5% GTA in 0.1 M PB for 4–6 h at 4 °C and post-fixed with 1% OsO_4_ in 0.1 M PB for 2 h at 4 °C, and then dehydrated in grades of alcohol and embedded in epoxy resin. Ultrathin sections were cut at 60 nm and stained with uranyl acetate and lead citrate. The specimens were observed under a TEM (JEM-1210; JEOL, Tokyo, Japan).

### Histoplanimetry

Digital images of more than 40 randomly selected glomeruli from each mouse were obtained at 400x magnification using BZ-X710 (Keyence). The size and number of total cells in each glomerulus were determined using PAS-H-stained sections. The number of B220^+^ B cells, CD3^+^ T cells, Iba1^+^ macrophages, WT1^+^ podocytes, and the immunopositive area for CD34+ capillaries and that for Nephrin^+^, Podocin^+^, or Synaptopdin^+^ reactions in the digital images of glomeruli were measured using immunofluorescence sections and a BZ-X Analyzer (Keyence). Images of 50 capillaries and their adjacent podocyte were obtained using mSEM examination and analysed using ImageJ (National Institute of Health, Bethesda, MD, USA) software to count EF and PFP per micrometre of length.

### Statistical analysis

The results were expressed as the mean ± standard error. For comparisons between healthy controls and diseased mice, a nonparametric Mann–Whitney U test (P < 0.05) was utilized. The Kruskal-Wallis test was used to compare between groups, and multiple comparisons were performed using Scheffe’s method when significant differences were observed (P < 0.05). The correlation between two parameters was analysed using Pearson’s rank correlation test (*p* < 0.01).

### Data availability

All data generated or analysed during this study are included in this published article and its Supplementary Information files.

## Electronic supplementary material

Supplementary information
