# Supplementary material for: Modified scanning electron microscopy reveals pathological crosstalk between endothelial cells and podocytes in a murine model of membranoproliferative glomerulonephritis
Source: Sci Rep. 2018 Jul 6;8:10276. doi: 10.1038/s41598-018-28617-1 (PMC6035194; doi:10.1038/s41598-018-28617-1)
Supplement: Supplementary file 1 — Supplementary information [file 41598_2018_28617_MOESM1_ESM.pdf]

Modified scanning electron microscopy reveals pathological crosstalk between endothelial cells and podocytes in a murine model of membranoproliferative glomerulonephritis

Authors: Md. Abdul Masum[1,2], Osamu Ichii[1,]*, Yaser Hosny Ali Elewa[1,3], Teppei Nakamura[1,4], Yuki Otani[1], Marina Hosotani[1]&Yasuhiro Kon[1]

Supplementary Table 1: Glomerular histopathology and clinical parameters

Parameters		Glomerular histopathology		Autoimmune indices	Renal function indices		
		Glo. Size (μm²)	Glo. Cell number	Anti-dsDNA ab (μg/mL)	uACR (μg/mg)	sCr (μg/dL)	sBUN (μg/dL)
**BXSB/MpJ (BXSB)**	***P***	3775.75 ± 113.23	34.16 ± 0.98	138.09 ± 21.70	144.93 ±15.52	0.32 ± 0.04	20.60 ± 1.49
**BXSB/MpJ-*Yaa* (Yaa)**	***P***	6241.24 ± 380.69*	72.75 ± 2.44*	712.20 ± 80.95*	801.89 ± 157.85*	0.95 ± 0.26*	74.62 ± 14.13*

Values = mean ± s.e. *: Significant difference from the control group, Mann-Whitney *U* test ($P < 0.05$). N = 4. Glo.: Glomerulus, Anti-dsDNA ab: Anti-double-strand DNA antibody, uACR, urinary albumin-to-creatinine ratio, sCr: serum creatinine and sBUN: serum blood urea nitrogen.

Supplementary Table 2: Correlation analysis for parameters examined in mice at 4 months of age.

Parameters	VEGF A⁺ Glo. area		PFP		Nephrin		Podocin		Synaptopodin		WT1⁺ Pod.	
	ρ	p	ρ	p	ρ	p	ρ	p	ρ	p	ρ	p
**CD34⁺ Glo. Cap.**	0.828*	0.011	0.429	0.289	0.303	0.466	0.773*	0.024	0.307	0.46	0.351	0.394
**EF**	0.481	0.288	0.405	0.319	-0.116	0.784	0.354	0.390	0.574	0.137	0.886**	0.003

*$p < 0.05$ and**$p < 0.01$, Pearson's rank correlation coefficient, N = 8. Glo.: Glomerulus, Cap.: Capillary, EF: Endothelial fenestration, Pod.: Podocyte, PFP: Podocyte foot process.

Supplementary Table 3: Summary of immunostaining conditions.

Parameters	B220	CD3	CD34	Iba1	Nephrin	Podocin	Synaptopodin	VEGF A	WT 1
**Antigen retrieval**	CB 105°C, 20 min	TB 105°C, 20 min	CB 105°C, 20 min	0.1% pepsin 37°C, 5 min	CB 105°C, 20 min	CB 105°C, 20 min	CB 105°C, 20 min	CB 105°C, 20 min	CB 105°C, 20 min
**Blocking**	10% NGS	10% NGS	10% NGS	10% NGS	5% NDS	5% NDS	5% NDS	5% NDS	5% NDS
**Primary antibody**	Rat polyclonal antibodies (Cedarlane, ON, Canada) 1:1000	Rabbit polyclonal antibodies (Nichirei, Tokyo, Japan) 1:200	Rabbit polyclonal antibodies (Abcam, Cambridge, UK) 1:400	Rabbit polyclonal antibodies (Wako, Tokyo, Japan) 1:2000	Rabbit polyclonal antibodies (1:500; IBL, Gunma, Japan)	Rabbit polyclonal antibodies (1:800; IBL, Gunma, Japan)	Mouse monoclonal antibodies (1:100; Fitzgerald, MA, USA)	Rabbit polyclonal antibodies (Abcam, Cambridge, UK) 1:200	Rabbit polyclonal antibodies (Santa Cruz, Dallas, TX,USA) 1:150
**Secondary antibody**	Rat anti-goat IgG (CaltagMedsystems, Buckingham, UK) 1:100 (Biotinylated)	Goat anti-rabbit (SABPO kit, Nichirei, Tokyo, Japan) 1:100 (Biotinylated)	Alexa Fluor 488-labeled donkey anti-rat IgG antibodies (1:500; Life Technologies)	Goat anti-rabbit IgG (SABPO kit, Nichirei, Tokyo, Japan) 1:100 (Biotinylated)	Alexa Fluor 546-labeled donkey anti-rabbit IgG antibodies (1:500; Life Technologies)	Alexa Fluor 546-labeled donkey anti-rabbit IgG antibodies (1:500; Life Technologies)	Alexa Fluor 546-labeled donkey anti-mouse IgG antibodies (1:500; Life Technologies)	Alexa Fluor 546-labeled donkey anti-rabbit IgG antibodies (1:500; Life Technologies)	Alexa Fluor 546-labeled donkey anti-rabbit IgG antibodies (1:500; Life Technologies)
CB: Citrate buffer, TB: Tris buffer, NGS: Normal goat serum, NDS: Normal donkey serum									

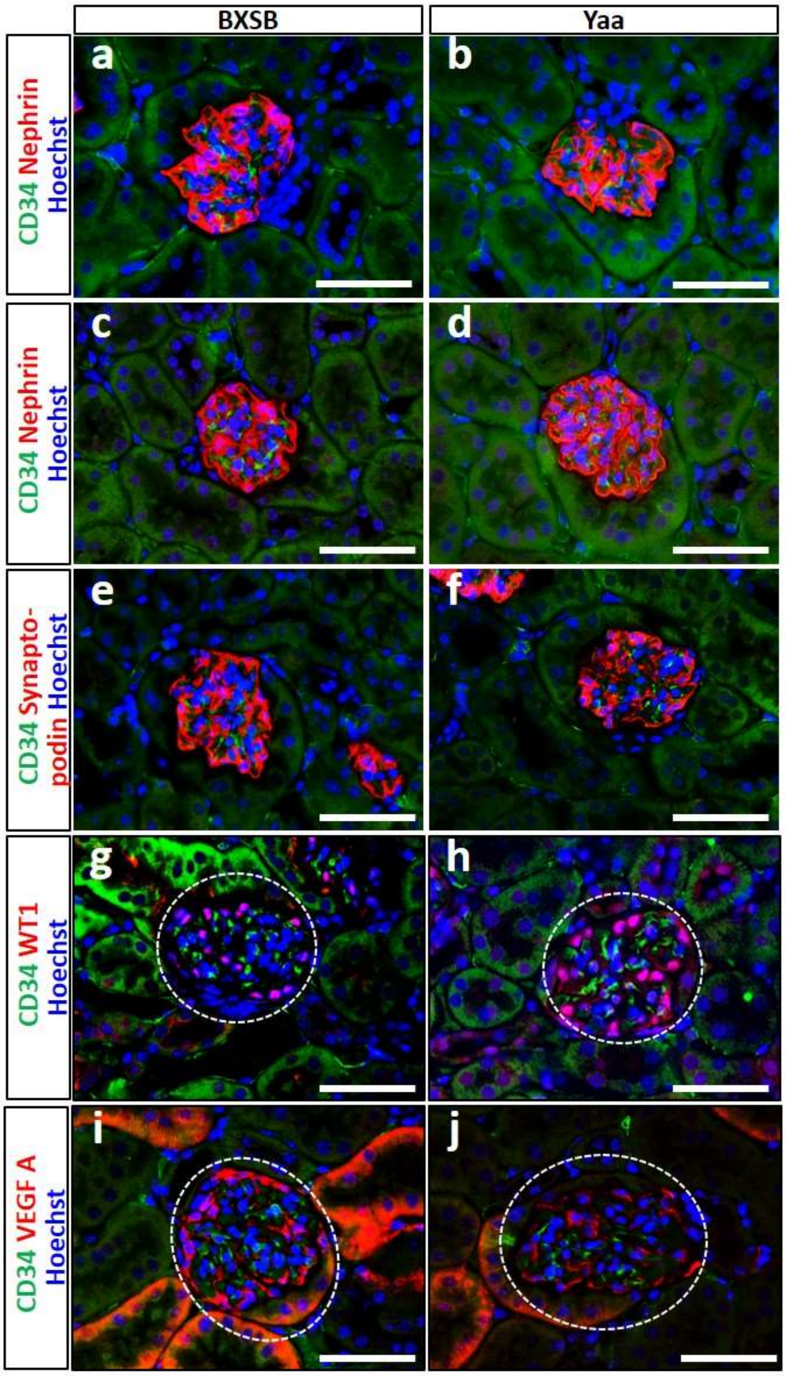

Supplementary Fig. 1: Glomerular capillary and podocyte function molecules.

(a-j) Endothelial cell marker (CD34) and podocyte function molecules (Nephrin, Podocin and Synaptopodin), immunofluorescence. CD34-, Nephrin-, Podocin-, Synaptopodin-, WT1-and VEGF A-immunopositive areas are clearly visible in BXSB (a, c, e, g and i) and Yaa (b, d, f, h and j) glomerulus at 3 months. Bars=50 µm.

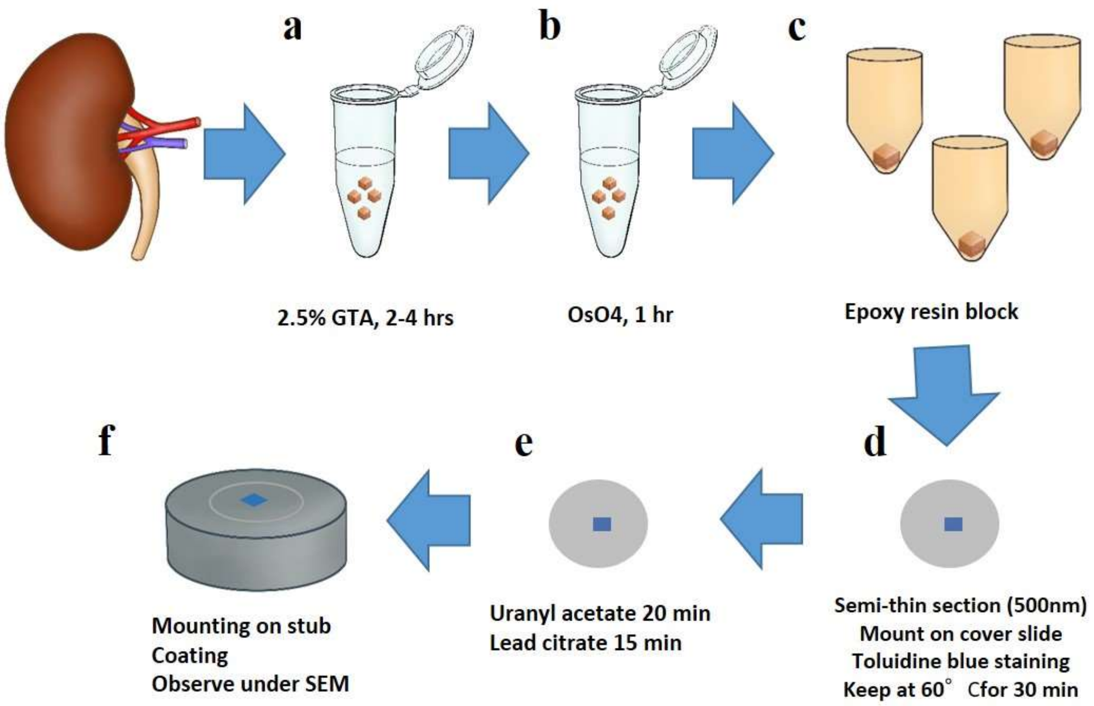

Supplementary Fig. 2: Illustration of mSEM method:

(a) Fixation of small pieces of kidney with 2.5% GTA for 2-4 hours

(b) Post fixation with 1% OsO_4 for 1 hour.

(c) Embedding in epoxy resin.

(d) Semi-thin sectioning, mounting on cover glass, staining with toluidine blue and drying.

(e) Staining with uranyl acetate and lead citrate.

(f) Mounting on the specimen stub, sputter coating and examination under SEM

GTA: glutaraldehyde, OsO_4: Osmium tetroxide and SEM: scanning electron microscope.
